# Hospital Disaster Preparedness in Iran: A Systematic Review and Meta-Analysis

**Published:** 2020-05

**Authors:** Jafar BAZYAR, Negar POURVAKHSHOORI, Hamid SAFARPOUR, Mehrdad FARROKHI, Hamid Reza KHANKEH, Salman DALIRI, Elham RAJABI, Vahid DELSHAD, Kourosh SAYEHMIRI

**Affiliations:** 1. Health in Emergency and Disaster Research Center, University of Social Welfare and Rehabilitation Sciences, Tehran, Iran; 2. Department of Health in Disasters and Emergencies, School of Public Health and Safety, Shahid Beheshti University of Medical Sciences, Tehran, Iran; 3. Department of Clinical Science and Education, Karolinska Institutet, Stockholm, Sweden; 4. Clinical Research Development Unit, Imam Hossein Hospital, Shahroud University of Medical Sciences, Shahroud, Iran; 5. Psychosocial Injuries Research Center, Ilam University of Medical Sciences, Ilam, Iran

**Keywords:** Iran, Disaster, Hospital preparedness, Systematic review, Meta-analysis

## Abstract

**Background:**

Disasters are increasing all over the world. Iran, is one of the high-risk countries in this regard; so it is unavoidable to prepare hospitals as vital centers when disasters happen. This study aimed to evaluation the hospital preparedness based on previous studies in Iran.

**Methods:**

A systematic review and meta-analysis by browsing through all articles published since 2006 to 2017, in English and Persian both languages were designed. Databases that we searched to, include Google Scholar, PubMed, Web of Science, Scopus, Medlib, Cochrane Library, Science Direct, Internationally and SID, Irandoc and Magiran, domestically. Two expert researchers investigated separately. Researchers used random and fixed effect models in the meta-analysis. Moreover, random and fixed effects model and meta-regression tests were applied by using STATA ver. 11. The *P*<0.05 was considered statistically significant.

**Results:**

Twenty-five studies with a sample size of 181 hospitals were introduced to the process of meta-analysis. Iranian hospital preparedness is 53%, totally, that is moderate. Preparedness in different categories is as follows: emergency services 62%, communication 57%, security 54%, education 57%, logistic 65%, human resources 52%, Management and command 64%, reception 43%, transfer and evacuation 44%, traffic 47%, non-structural safety 57%, and structural safety 49%.

**Conclusion:**

Hospital preparedness is moderate in Iran. Optimal management of existing resources and the use of Update technologies in the field of hospital services be directed towards improving the preparedness of hospitals for disasters.

## Introduction

Natural hazards have long been affecting human societies and have caused abundant physical harm and death of many human beings. Today, these disasters are rising in the world and have had a tremendous impact on human life and health, so far as their devastating and destructive impact have disrupted society’s ability to meet its basic needs and caused injury, disability and death of many people. Disasters have also imposed economic costs to the government and nations through damaging homes and properties of the people. Over the past thirty years, disasters have doubled in the world, and the rate of damage and personal injuries has tripled, which shows the importance of taking actions to reduce the impact of disasters on human societies (1–3).

According to global reports in 2016, Iran is not among the 10 most disaster-prone countries in the world. Moreover, the trend of physical damage and deaths caused by disasters in Iran has decreased between 1995 and 2014 (4, 5). However, in the recent years, an average of 253 hazards is occurring in Iran per year. Obviously, disasters, depending on the type, extent, frequency, and density of the population facing with the hazards, lead to small and big catastrophes that are sometimes really difficult to be recovered (6). One of the main attributes of a natural hazards is its unpredictability. Therefore, preparedness is the only way to deal with this phenomenon, which can subsequently prevent or reduce the amount of damage caused by it. After the occurrence of emergencies and disasters, cross-regional services are necessary to respond and compensate the damages imposed, among which health and medical systems are the most basic units in providing services to the injured people (7). During the first 24 to 48 h after a disaster, the greatest need for the health care is felt, so that 85%–95% of the survivors need relief and health care, during the first 24 h. Therefore, all hospitals should be well prepared to manage and provide services, so that they can promptly and efficiently provide timely health services to the injured patients to reduce mortality and increase the number of survivors (8, 9). Thus, hospitals should be prepared to deal with disasters, before they occur.

In a study in Italy, which evaluated the preparedness level of hospitals against disasters using the WHO checklist, the preparedness of the hospitals is less than the level recommended by the WHO (10). In Tanzania, which evaluated the preparedness of 25 hospitals against disasters, the hospital preparedness in all areas was estimated to be between 20% and 60% (11). The level of hospital preparedness was estimated in the 27 EU member states at 68%, which was at an acceptable level (12).

Regarding the probability of the occurrence of these hazards, especially in Iran, and the increasing occurrence rate of these disasters and their related consequences, the critical role of health services before, during and after disasters is more felt, and the proper preparation of service providers seems essential in all of the hospitals. So far, there has been no comprehensive study on the overall assessment of the hospital preparedness against disasters in Iran. Thus, the present study aimed to evaluate the preparedness of the Iranian hospitals against disasters to provide comprehensive information on the topic, through which one can take appropriate measures for improving the preparedness level of the hospitals of the country against disasters.

## Methods

### Search strategy and selection criteria

A systematic review and meta-analysis was conducted to identify the hospital preparedness against emergencies and disasters in Iran. The review was conducted following PRISMA guidelines (13). The results of this study were based on the articles published in Persian and English language journals. In this research, all articles published from the beginning of 2006 through the end of 2017 were selected through the Medlib, Scopus, Web of Science, PubMed, Cochrane Library, Science Direct, Google scholar, Irandoc, Magiran, and SID databases. All the articles with medical subject headings (MeSH) keywords of hospital preparedness for disasters, emergency services, reception, security, education, support, structural preparedness, non-structural preparedness, and management and commanding were the search keywords used in isolation or in with the operator “OR” vs “AND”.

### Quality assessment

For qualification assessment of the articles, the Strengthening the Reporting of Observational Studies in Epidemiology (STROBE) checklist was applied (14). This checklist contains 22 different parts and the score allocation for this checklist is based on the importance of each part, and the least score for article qualification is 15 out of 33. In this study, a score of 20 and above was acceptable (15).

### Data extraction, analysis, and synthesis

First, all articles assessing the hospital preparedness against disasters were collected and, upon completion of the search, a list of the abstracts was also prepared. After concealing the profile of the articles, such as the name of the author, journal, etc., the full texts of the articles were given to two trained researchers to review. Each article was reviewed by the two individuals, independently. If the articles were rejected by the two reviewers, the reason was also mentioned and in case of disagreement between them, the article was judged by a third reviewer.

The required data were extracted using a pre-prepared checklist including the sample size, study location, study time, type of study, hospital preparedness for disasters, emergency services, reception, security, education, logistics, structural- and non-structural preparation, management and commanding as well as the main elements of the hospital preparedness introduced by the health assessment tools (16), and communicated by the Ministry of Health. Accordingly, the relative frequency of the hospital preparedness indices against disasters was investigated.

All Persian and English articles published on hospital preparedness for emergencies and disasters, conducted in the hospitals of Iran and those which included the required information were considered for further evaluation. The studies that did not examine the hospital preparedness criteria according to the existing standards or those which discussed hospital emergency responses to disasters, but lacked the data required for estimating the relative frequency were excluded from the study. In addition, interventional, qualitative or series studies were excluded from the study.

**Fig. 1:**
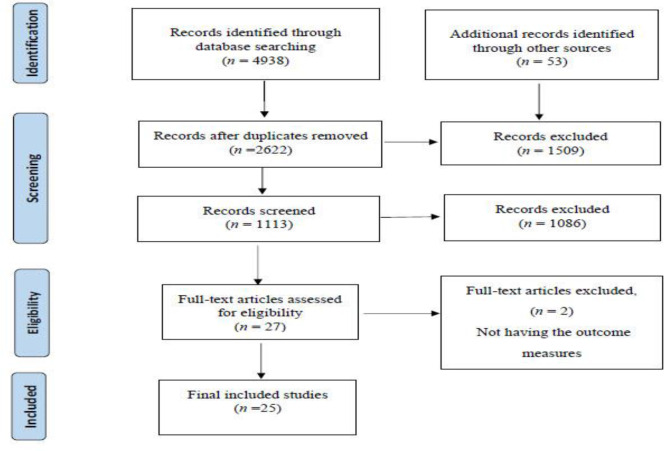
Results of the systematic literature review

There were 135 articles related to hospital preparedness for emergencies and disasters, of which 36 and 59 were further excluded due to repetitiveness and irrelevance to the study, respectively. After reviewing the abstracts of the articles, 12 articles lacking required information and 3 articles due to the uncertainty of the studied population, inadequacy of the surveyed population and not using the standard definition for the indicators of the hospital preparedness were excluded, too. Finally, 25 articles meeting the required criteria entered into the meta-analysis procedure (Fig. 1).

To combine the results in heterogeneous studies, random-effects model was used and in the homogeneous studies, the fixed-effects model in meta-analysis was deployed. To investigate the heterogeneity of the data, *I^2^* and Cochrane tests were used. Publication bias was controlled by Egger test and Funnel plot and data analysis was performed using STATA ver. 11 software. The significance level was considered to be 0.05.

## Results

**Fig. 2:**
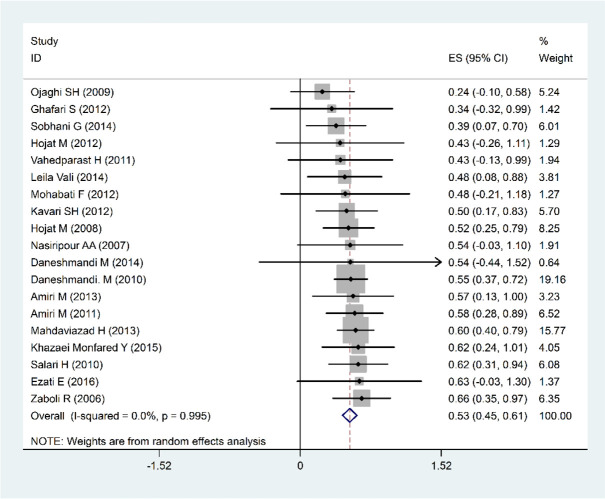
Forest plots of Percentage of relative frequency of Total Hospital Preparedness for Disasters and confidence interval 95% based on a Fixed effects model in the meta-analysis. The midpoint of each segment and the segment estimating the Percentage of relative frequency and confidence interval 95% in every study show. Mark Diamond overall percentage of relative frequency based upon the results of the meta-analysis of studies shows

Overall, 25 articles with a sample size of 181 Iranian hospitals that were reviewed between 2006 and 2017 were included in this study. All the studies were descriptive-analytic; the specifics of the articles under consideration are presented in Table 1. Nineteen studies with a sample size of 151 hospitals had an overall hospital disaster preparedness index. The overall preparedness of the Iranian hospitals against disasters was estimated at 53% (45%–61%: 95% confidence interval), which is moderate (Fig. 2).

**Table 1:** General chaFracteristics of the studied articles that were eligible for the meta-analysis

***Author***	***Study location***	***Year of study***	***number of samples***	***Study Type***	***Abstract finding***
Sobhani G(17)	Bandar Abbas	2014	9	Cross-sectional	The overall level of preparedness against disasters was 38.6%. They were at a poor level in the areas of reception 31.4%, evacuation 28.1%, traffic 33.3%, security 34.6%, communication 30.6%, human resources 38.6%, and commanding and management 20.1%.
Amiri M(18)	Semnan	2011	10	Cross-sectional	Average preparedness of programming support for vital services was 80%, for disasters management programs in hospital was 65%, for programming for environmental health activities against disasters was 56.2%, for security of equipment and hazardous material was 64.2%, for programming to reduction in structural dangers was 43.8%, for evacuation and field treatment was 49.5%, and average score for hospital educational planning to deal with disasters was 42.2%.
Maleki M(19)	Tehran	2007	10	Cross-sectional	In hospitals, security preparedness was estimated at 69.9%.
Ezati E(20)	Kermanshah	2016	2	Cross sectional	Hospital preparedness for emergencies 73.3%, admission 31.65%, security 72.25%, training 88.2% and support 73.5%.
Danesh mandi M(8)	Tehran	2014	1	Cross-sectional	Amount of preparation of the study hospital in the department’s traffic 33.3%. In the category of weak and units of acceptance, communication, education and transport, discharge respectively, with an average 41.7%, 50%, 41.2%, 46.7% was assessed average level Other areas include the emergency department, security, support, staffing and management, respectively, with an mean 66.7%, 64.7%64.3%61.9% and 68.2% which were rated as good.
Vali L(21)	Tabriz	2014	6	Cross-sectional	There was an average level of preparedness in the fields of emergency (54%), support (57%) and traffic (58%) while they were in a readier condition than other fields. Scores concerning human resources (77%) and organization and structure (66%) represent a satisfactory level of preparedness.
Mohabati F(22)	Zabol	2012	2	Cross sectional	The hospitals preparation rate in planning (2.27%), cleaning (30%), emergency (42.85%), logistic services (48.61%), patients transfer and discharge (38.33%), traffic controls (77.26%), communications (31.57%), health (35.71%) and safety (47.61%).
Hojat M(23)	Jahrom	2012	2	Cross sectional	Hospital preparedness for transfer and discharging 10.27%, acceptance 31.66%, communications 34.16%, management 38.33%, urgencies 53.8%, traffic 36.66%, human resources 47.3%, security 50.41%, and support 41%.
GhanbariV(24)	Tehran	2012	2	Cross-sectional	Hospital preparedness for acceptance 75%, transfer and discharging 25%, security 35%, training 43.75% and support 17.64%.
Shafaght T (25)	Shiraz	2010	9	Cross-sectional	Overall, the average relative preparedness of coping with unexpected hazards in the hospitals was 62.3%.
Daneshmandi M(26)	Iran	2010	30	Cross-sectional	The average preparedness of different parts of hospitals, including reception, security, discharging and transfer, manpower, communications, traffic, emergency, training, support and management, totaled 21%, 45%, 49%, 44.5%, 54%, 49%, and 64%.
Ameriun A(27)	Iran	2010	3	Cross-sectional	From the total 11 dimensions studied in the selected hospitals, hospital A with the mean score of 87.1%, hospital B with mean score of 77.59% and Finally hospital C by mean score of 70.01% had the preparedness of confrontation with disasters. In general, the average of preparedness in three studied hospitals was 78.23%.
Ojaghi SH(28)	Kermanshah	2009	6	Cross-sectional	The average overall preparedness rate for all hospitals was 23.8%.
Hojat M(29)	Tehran	2008	13	Cross sectional	Hospital preparedness for admission, transfer and discharging, emergency, traffic, communications, manpower and management was 38.32%, 39.63%, 48.20%, 52.33%, 52.14%, 43.8% and 48%.
Nasiripour AA(9)	Kermanshah	2007	3	Cross-sectional	The overall preparedness of the hospitals was 53.6%, in education 50.8% and in the 61.8% area.
Zaboli R(30)	Tehran	2006	9	Cross-sectional	Hospital preparedness for training was 40%, management and command 37.6%, and overall safety was 66%.
Shojaei P(31)	Tehran	2009	4	Cross-sectional	The hospital preparedness in field of security was 88.4%, supplies and equipment 66.6%, evacuation 64.2%, communication, 63.2%.
Vahedparast H(32)	Bushehr	2011	3	Cross-sectional	The hospital preparedness in traffic base was very poor with mean number of 19.04±16.10 evaluation of security; education and management domain with mean number 35.29±26.52, 38.65±19.46, 36.36±24.05, respectively were poor.
Amiri M(33)	North of Iran	2013	5	Cross-sectional	The average score of managers’ awareness of the disaster confronting Preparedness status was 41.89±9.12 and hospital’s preparedness to confront disasters was 56.88±5.12 which show a mediocre level in all hospitals studied.
Kavari SH(34)	Shiraz	2012	9	Crosssectional	Hospital preparedness for training 50%, structural preparation 50%, and non-structural 50%.
Mahdaviazad H(35)	Shiraz	2013	24	Cross-sectional	The scores for preparedness of ICS, communication, surge capacity and human resources was 73.9%, 67.3%, 49%, and 52.6%.
Khazaei Monfared Y(36)	Qazvin	2015	6	Cross-sectional	Functional, structural, and nonstructural safety scores were evaluated as 61.58% (average safety), 64.44% (average safety), and 61% (average safety), respectively. General preparedness of the hospitals we studied were 62.34%, an average safety level.
PartouiShayan Z(37)	Qazvin	2014	6	Cross-sectional	The emergency preparedness facilities of the studied educational centers were evaluated for dealing with disasters of 70.72%.
Mohamadi S(38)	Kermanshah	2017	3	Cross-sectional	Emergency preparedness was 76.65, traffic was 68.8%, communication was 70.8% and security was 79.6%.
Mirzaei F(39)	Ilam	2014	4	Cross-sectional	Imam highest scores in all three functional, non-structural and structural and the lowest score acquired in the performance section of the Patients in the non-structural and structural and Kosar is related to Taleghani Hospital.

The highest rate of hospital preparedness was reported for Shiraz (2010) (25) and Kermanshah (2016) (38) with 78% and 77% of preparedness, respectively, and the lowest level of hospital preparedness was reported for Zabol (2012)(22) which was equal to 43%.

**Fig. 3:**
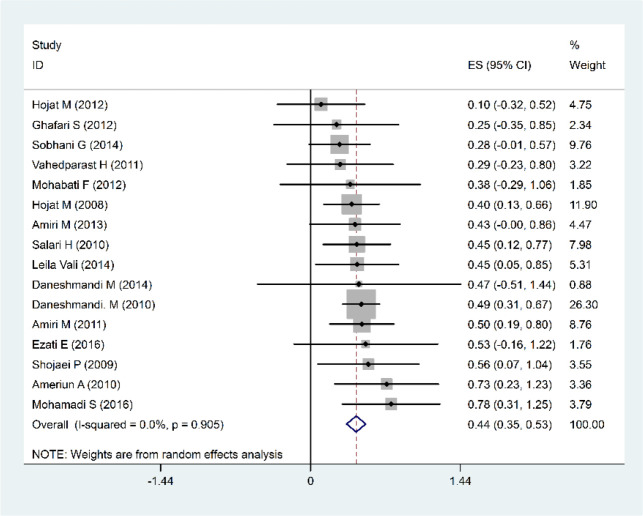
Forest plots of Percentage of relative frequency of Hospital Preparedness. In the field of Transfer and Evacuation for Disasters and confidence interval 95% based on a Fixed effects model in the meta-analysis. The midpoint of each segment and the segment estimating the Percentage of relative frequency and confidence interval 95% in every study show. Mark Diamond Overall Percentage of relative frequency based upon the results of the meta-analysis of studies shows

**Fig. 4:**
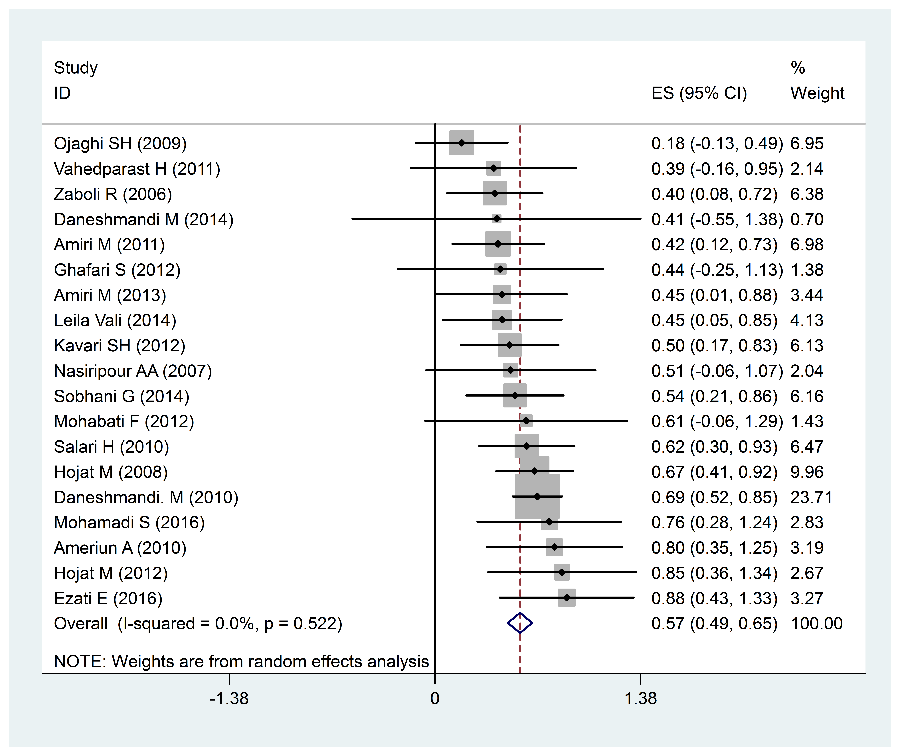
Forest plots of Percentage of relative frequency of Hospital Preparedness in the field of Education for Disasters and confidence interval 95% based on a Fixed effects model in the meta-analysis. The midpoint of each segment and the segment estimating the Percentage of relative frequency and confidence interval 95% in every study show. Mark Diamond Overall Percentage of relative frequency based upon the results of the meta-analysis of studies shows

Based on the meta-analysis of 16 studies conducted from 2008 to 2016 with a sample size of 104 hospitals, the hospital preparedness of the transfer and evacuation index was 44% (35%–53%: 95% CI), suggesting that the rate of the hospital preparedness in this area is less than average (Fig. 3). As far as education index is considered, 19 studies (2006–2016) with a sample size of 127 hospitals were evaluated, showing a relative frequency distribution of 57%, which indicates that hospital preparedness in this category is moderate, as well (Fig. 4).

In the field of transportation, the highest level of preparedness was reported for Kermanshah (78%) (39) and the lowest level was reported for Jahrom (10%)(23). Morever, in the field of education, the highest level of preparedness was reported for Kermanshah (88%) and the lowest for Bushehr (39%)(32).

In general, of 25 studies from 2006 to 2016, with the sample size of 181 hospitals, we can present the following statistics for different indices of the hospital preparedness against disasters: emergency services (62%), communications (57%), security (54%), logistics (65%), management and commanding (64%), human resources (52%) and non-structural safety (57%), which were all at an intermediate level. Other indices are calculated as follows: reception (43%), traffic (47%) and structural safety (49%) which were at a low level (Table 2). In the case studies conducted in the afore mentioned years, the most desirable preparedness indicators including emergency services, communications, security, logistics, management and commanding, human resources, traffic, reception, structural safety, and non-structural safety belonged to Kermanshah (75%), Qazvin (76%), Kermanshah (80%), Semnan (80%), Tehran (91%), Shiraz (72%), Zabol (77%), Kermanshah (75%), Shiraz (64%) and Semnan hospitals (64%). On the other hand, the weakest preparedness indices belonged to Tabriz (30%), Bandar Abbas (31%), Bandar Abbas (35%), Tehran (18%), Bandar Abbas (20%), Bandar Abbas (39%), Bushehr (14%), Tabriz (30%), Northern Iran (41%) and Tehran hospitals (33%).

**Table 2:** Analyzing hospital preparedness against emergencies and disasters using a meta-analysis

***Variable***	***Number of study***	***Number of hospitals***	***Mean Percentage of frequency %***	***CI 95%***
*Emergency services*	11	80	62	(52–73)
*Reception*	12	76	43	(29–56)
*Transfer and evacuation*	16	104	44	(35–53)
*Traffic*	11	80	47	(36–57)
*Communications*	15	117	57	(49–66)
*Security*	15	102	54	(45–64)
*Education*	19	127	57	(49–65)
*Logistic*	15	121	65	(57–73)
*Human resources*	13	89	52	(42–62)
*Commanding and Management*	17	136	64	(53–74)
*Structural*	5	32	49	(32–67)
*Nonstructural*	5	32	57	(40–74)
*Total preparedness*	19	151	53	(45–61)

**Fig. 5:**
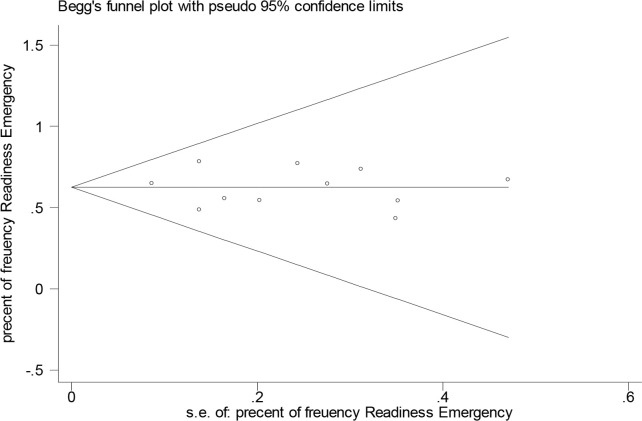
Funnel chart of the frequency Preparedness Emergency among the evaluated studies

In order to study the publication bias in this research, the funnel diagram was used. Accordingly, given the symmetry of the chart, there has been no publication bias in this study (Fig. 5).

Using the Egger test, a statistically significant level (*P*=0.77) was obtained, this relationship is not significant, and there is no publication bias in this study. Finally, there may be significant statistical correlations between the year of the study and hospital preparedness against disasters, this relationship was investigated using meta- regression.

**Fig. 6:**
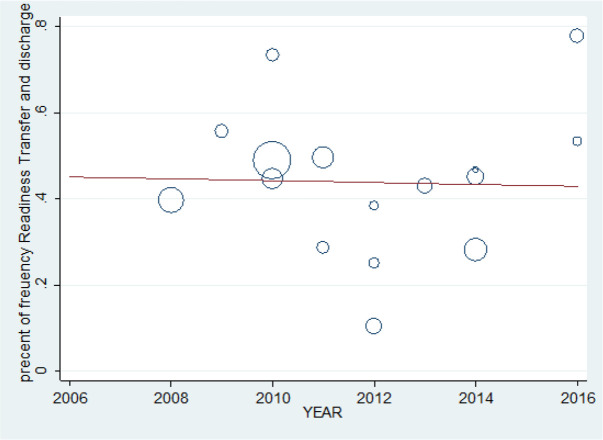
Meta-regression chart of percent of frequency Preparedness Transfer and Discharge upon the year of study

At the end, according to the slope of the meta- regression curve, it was found that there was no significant correlation between the increase or decrease in the percentage of the hospital preparedness for disasters and increase of the year of the study (Fig. 6).

## Discussion

Based on the findings, the overall preparedness of the Iranian hospitals against disasters was moderate. In Yemen, the rate of the hospital preparedness for disasters was 46.6% (40). The level of preparedness of rural American hospitals was estimated at an intermediate level (78%) (41). In Italy, the hospital preparedness in the three parts was estimated at a high level, which is consistent with our study (10). In Jordan, the level of hospital preparedness for disasters was assessed at a poor level (42). Only 22% of the hospitals in the United States were well prepared to face with disasters (43). The preparedness of the Iranian and Swedish hospitals for disasters in a comparative way, hospitals in Sweden had an excellent preparedness against disasters (44). Appropriate measures should be taken in the Iranian hospitals to improve the performance of the various fields of hospital preparedness against disasters, in order to effectively reduce the mortality rate and physical damage during a disaster.

Our findings showed that hospital preparedness against disasters in the areas of education and human resources was at a moderate level (50%–80%) but in the areas of reception were at a weak level (<50%). In Iran, the hospital preparedness for disasters in human resources was moderately estimated and the score was high as far as education were evaluated (26). Education was considered as a pivotal component of disaster preparedness and damage prevention during a disaster. Improving education in the field of disaster response can also improve the performance of personnel in other areas of the hospital preparedness (45).

Moreover, in the present meta-analysis, the hospital preparedness in the areas of emergency services, communications, management and commanding and logistics was at a moderate level (50%–80%). Pagnin et al. estimated the emergency preparedness rate for Italian hospitals modestly and stated that about 65% of the hospitals were prepared to deal with disasters (46). In Italy, the hospital preparedness for disasters in the fields of management, communications, emergency services and logistics was modest, but the score for these indicators was lower than the global average (10). In the study conducted on 30 hospitals in Iran, the hospital preparedness for disasters in emergency services, transfer and evacuation was moderately estimated and the score was high as far as logistics, management and commanding were evaluated (26). Comparatively, the estimated level of preparedness in other studies seems to be less than our results. The preparedness of the Iranian hospitals for reception and traffic was estimated at poor and intermediate levels, respectively (26). This fact can be explained through the improvement of the hospital performance over the recent years in terms of hospital preparedness for disasters, or using a different kind of questionnaire in these studies. Standard tools and questionnaires are used to measure hospital preparedness against disasters more accurately (47).

The hospital preparedness for security and non-structural safety was at a moderate level but in the areas of structural safety is at a weak level. The hospital preparedness for disasters in security was moderately estimated (26). In Caribbean, 80% of the hospitals had a moderate level of safety, 18% of the hospitals had a low level of safety, and only 2% were completely safe (48). In Moldova, 24.6% of the hospitals had a high level of safety. Accordingly, 67.2% and 8.2% had moderate safety and poor safety, correspondingly (49). Examination of the safety of the hospitals in Iran, estimated the safety of the hospitals at 32.4%, which was at a weak level. As reported in this study, the safety level of the Iranian hospitals was poor and moderate in 54% and 46% of the cases, respectively. None of the hospitals had high levels of safety. The estimated score for non-structural and structural safety was 36%, indicating that they were also at a weak level, which is consistent with the results of our study (50).

In general, to improve the performance of hospital preparedness against disasters in a country, it is necessary to improve the performance of all fields. Education is one of the main pillars for improving disaster management, which should be improved through training programs. The emergency unit needs planning in the field of equipment provision, job clarifications, organization of the members of the triage team and the coherence of the organizational structure of the sector prior to the occurrence of unexpected hazards. In the field of traffic, transfer and evacuation, it is necessary to consider the space for parking, training of the human resources and provision of the necessary equipment. It is also important to specify the proper route for the patients to depart from the sections to the open space, provide suitable equipment and facilities for disabled patients to exit and predetermine the right place to accommodate patients in the hospital. As far as structural and non-structural safety is considered, hospitals should be reconstructed and renovated in order to eschew serious damage, when disasters occur. As for management and commanding, logistics, communication and human resources indicators, we can develop specific guidelines and programs in the field of management structure, specification of the duties of the managers and also implementation of the regular education programs, which can improve the performance of these categories. Identifying notification processes, describing communication tasks, using early warning system, providing equipment, personnel and executive instructions in the field of security and logistics are other factors that can be applied to improve the performance of other fields, which ultimately can lead to the improvement of the overall performance of hospitals in dealing with disasters.

### Implications for Policy & Practice

- The overall preparedness of Iranian hospitals to deal with disasters and emergencies was moderate.
- To improve the hospital’s Preparedness to deal with disasters and emergencies in a country, it is necessary to improve the performance of all areas.
- By adopting appropriate measures in the fields of education, equipment and human resources, as well as the reconstruction and rehabilitation of hospitals, we can improve the preparedness of the hospitals.

## Conclusion

The level of preparedness of hospitals in Iran was in moderate level, it is recommended that by conducting Exercise and training programs for the hospital staff, proper management of resources and training in the field of efficient use of existing equipment in the encounter of disaster to improve personnel performance and better preparedness of hospitals in Iran to deal against disasters.

## Ethical considerations

Ethical issues (Including plagiarism, informed consent, misconduct, data fabrication and/or falsification, double publication and/or submission, redundancy, etc.) have been completely observed by the authors.
